# A medical decision support system for predicting the severity level of COVID-19

**DOI:** 10.1007/s40747-021-00312-1

**Published:** 2021-03-04

**Authors:** Mohsen Abbaspour Onari, Samuel Yousefi, Masome Rabieepour, Azra Alizadeh, Mustafa Jahangoshai Rezaee

**Affiliations:** 1 Faculty of Industrial Engineering, Urmia University of Technology, Urmia, Iran; 2 Pulmonary Department, Urmia University of Medical Sciences, Urmia, Iran; 3 Department of Internal Medicine, Urmia University of Medical Sciences, Urmia, Iran

**Keywords:** COVID-19, Medical decision support system, Severity level prediction, Evidence-based paired relationships, Data-driven Bayesian network, Fuzzy cognitive map

## Abstract

The main assay tool of COVID-19, as a pandemic, still has significant faults. To ameliorate the current situation, all facilities and tools in this realm should be implemented to encounter this epidemic. The current study has endeavored to propose a self-assessment decision support system (DSS) for distinguishing the severity of the COVID-19 between confirmed cases to optimize the patient care process. For this purpose, a DSS has been developed by the combination of the data-driven Bayesian network (BN) and the Fuzzy Cognitive Map (FCM). First, all of the data are utilized to extract the evidence-based paired (EBP) relationships between symptoms and symptoms’ impact probability. Then, the results are evaluated in both independent and combined scenarios. After categorizing data in the triple severity levels by self-organizing map, the EBP relationships between symptoms are extracted by BN, and their significance is achieved and ranked by FCM. The results show that the most common symptoms necessarily do not have the key role in distinguishing the severity of the COVID-19, and extracting the EBP relationships could have better insight into the severity of the disease.

## Introduction

**Fig. 1:**
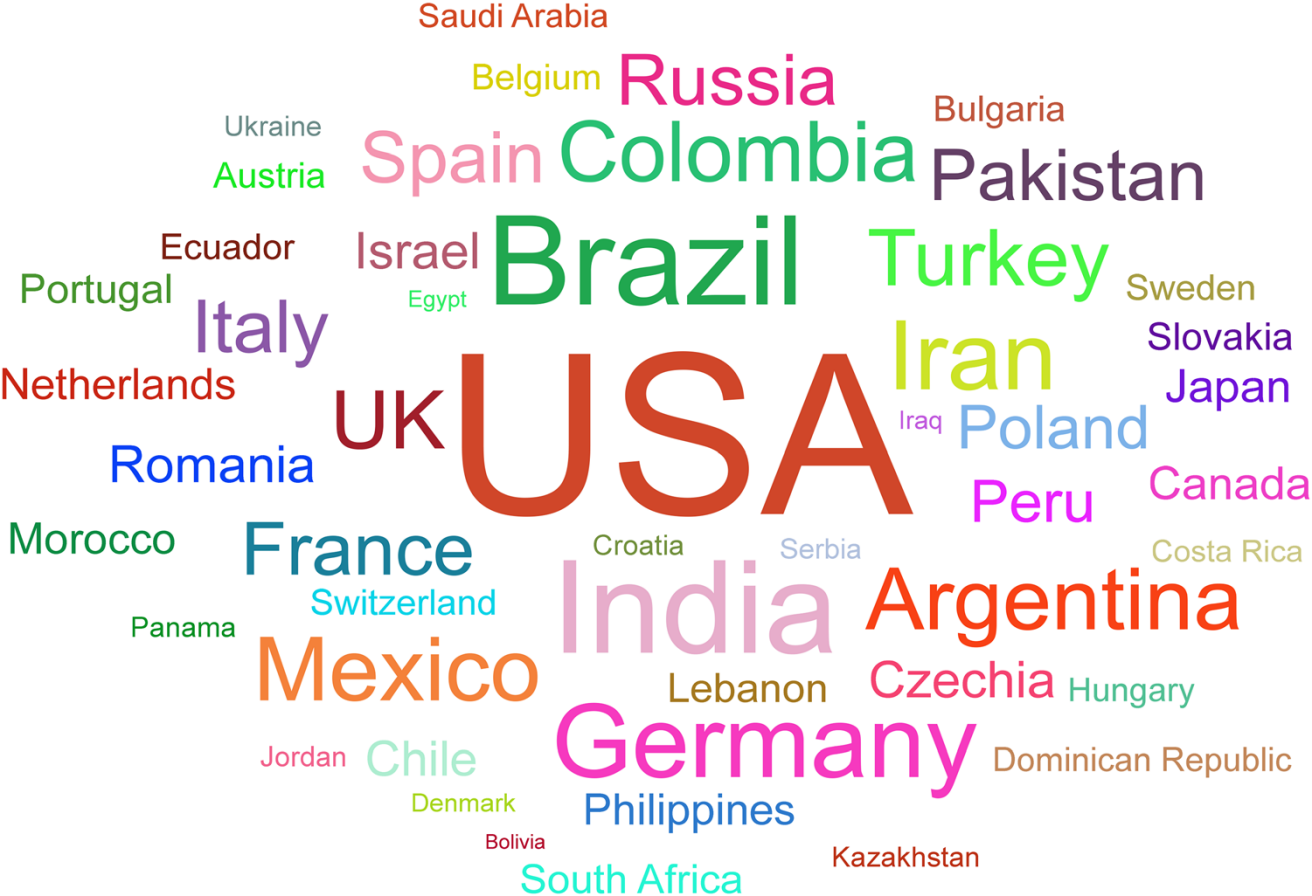
Total cases of COVID-19 in countries with the highest number of patients per 1 million

**Fig. 2:**
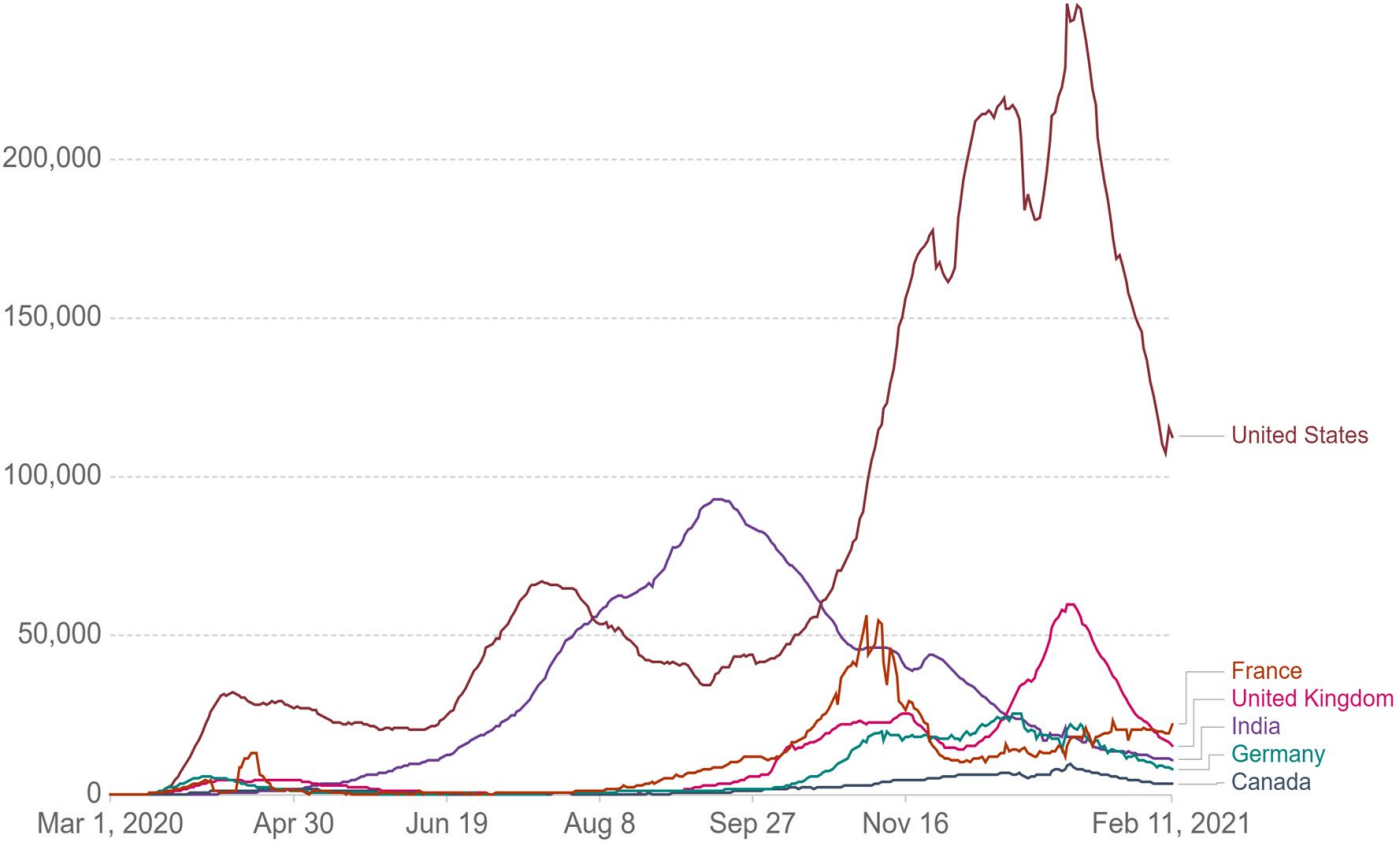
Daily new confirmed cases

Since December 2019, a couple of “unknown viral pneumonia” stems from a local Seafood Wholesale Market were reported in Wuhan city, the capital of Hubei province in China [1]. The Coronavirus Disease 2019 (COVID-19) is taken into account by a family of the deadly Severe Acute Respiratory Syndrome (SARS) and the Middle East Respiratory Syndrome (MERS) Coronaviruses [2, 3]. In just 2 months, the virus spread from Wuhan to the whole of China and 33 countries [1]. Consequently, the World Health Organization (WHO), on 11 Mar. 2020, announced COVID-2019 caused by SARS-CoV-2 to be a pandemic and public health emergency of an international outbreak [4]. To date, 12 Feb. 108,354,740 confirmed cases have been recorded worldwide that unfortunately 2,380,451 have passed away, and 80,405,951 of them have been recovered [5]. Figure 1 represents the total number of confirmed cases in countries with the highest number of patients per one million populations [5]. Figure 2 demonstrates the daily new confirmed cases [6].

COVID-19 infection causes systemic and respiratory disorders in the patient. Systemic disorders include fever, cough, fatigue, sputum production, headache, hemoptysis, acute cardiac injury, hypoxemia, dyspnoea, lymphopenia, and diarrhea. Respiratory disorders consist of rhinorrhoea, sneezing, sore throat, pneumonia, ground-grass opacities, RNAemia, and acute respiratory distress syndrome [7]. Fever, cough, and fatigue are the most common symptoms of COVID-19 at the early stages of the epidemic’s advent. In contrast, later symptoms include sputum production, headache, hemoptysis, diarrhea, dyspnoea, and lymphopenia [7]. The disease’s detection at the early stages is critical, because there are no specific therapeutic drugs for COVID-19. After detecting the infected patient’s symptoms, he/she should be isolated immediately from the healthy population [1]. Until now, real-time reverse transcription-polymerase-chain-reaction (RT-PCR) has been developed to assay COVID-19 in clinics [1]. RT-PCR still is the reference standard to make a definitive diagnose of COVID-19 infection. Still, the high false-negative rate and its unavailability in the early stage of the disease confined quick diagnosis of infected patients [1]. Much research has been conducted to fight COVID-19 in various science domains in a limited time based on the provided information, which one of them is Artificial Intelligence (A.I) tools.

Srinivasa Rao and Vazquez [8] proposed that using Machine Learning (ML) algorithms for identifying a person under investigation for the COVID-19 infection with a mobile phone-based web survey could identify the high-risk cases and quarantined earlier, and subsequently decreasing the chance of spread. Chest computed tomography (CT) can recognize the early phase of lung infection and prompt larger public health surveillance and response systems [1]. Li et al. [9] constructed a convolutional neural network (CNN) to identify community-acquired pneumonia on chest CT exams and could successfully distinguish COVID-19 cases from community-acquired pneumonia and other non-pneumonic lung diseases. The emergency management and infection control teams at the radiology department of West China Hospital formulated various measures to battle COVID-19, which protected all of the staff from COVID-19 risk: the reconfiguration of the radiology department, personal protection and training of staff, examination procedures for patients suspected of or confirmed with COVID-19 as well as patients without an exposure history or symptoms, and scanning persons with suspected or confirmed COVID-19 infection in the determined fever-CT unit [10]. Besides routine therapy, Xu et al. [11] prescribed tocilizumab to their patients and analyzed changes of clinical manifestations, CT-scan images, and laboratory trials retrospectively. Their results claim that the fever returned to normal within a few days, and all other symptoms improved significantly, and any obvious adverse reactions were observed. Tang et al. [12] modeled and trained a random forest to evaluate the severity of patients suffering from COVID-19 based on the chest CT images’ quantitative features. Farid et al. [13] combined conventional statistical and ML to extract features from CT images and then classified extracted features by a hybrid classifier system based on Naive Bayes.

Taiwan’s reactions against COVID-19 are phenomenal. Its response against COVID-19 includes three phases: (1) big data analytics: by gathering national health insurance database and integrating with immigration and customs database; (2) implementing new technology: using QR code scanning and online reporting to classify travelers’ infectious risks based on-flight origin and travel history in the past 14 days; (3) proactive testing: to amplify the COVID-19 case finding [2, 3]. Karar et al. [14] using X-ray scans proposed computer-aided diagnosis (CAD) systems based on cascaded deep learning (DL) classifiers for COVID-19. A similar study, analyzing chest X-ray images, has been conducted by Shankar and Perumal [15] for COVID-19 diagnosis and classification using a novel hand-crafted with DL feature-based fusion model. Elaziz et al. [16] based on the extracted features from the COVID-19 chest X-ray images using new fractional multichannel exponent moments (FrMEMs) exploited a modified manta-ray foraging optimization based on differential evolution to select the most significant features. de Moraes et al. (2020) implemented support vector machines (SVM) to extract features through multi-level thresholding from chest X-ray radiography images for early detection of COVID-19 cases. Laguarta et al. [17] developed a speech processing framework based on the CNN architecture for COVID-19′s patients’ cough recordings to discriminate them accurately. Wang et al. [2, 3] utilized linear discriminant analysis (LDA) for investigating the characteristics and rules of hematology changes in COVID-19 patients. Moreover, clinical and laboratory patients’ test results were analyzed, and different hematological parameters fitted using LDA.

Given the importance of the pandemic, the current study attempts to propose an intelligent self-assessment decision support system (DSS) based on the combination of Bayesian network (BN) and the fuzzy cognitive map (FCM) to utilize the key symptoms for distinguishing the severity of the disease between confirmed cases. In self-assessment systems, patients can obtain a general evaluation of their disease by reporting information about their symptoms. For designing an efficient self-assessment DSS, symptoms are analyzed both entirely and in severity levels. First, BN is implemented to extract evidence-based paired (EBP) relationships and symptoms’ impact probability for distinguishing severities for the whole data in various scenarios. To put it precisely, BN is applied to extract the EBP relationships between triple severity levels to develop FCM. BNs have various advantages: the ability to combine different knowledge sources, the capacity to handle small and defective datasets, and the availability of a wide range of validation methods besides data-driven validation methods [18]. Hence, it is a very powerful method to implement in the healthcare realm. This method has been applied in diagnosing breast cancer [19], Alzheimer’s disease [20], and erythematous-squamous diseases [21]. In the second stage, in triple severities, the FCM is used to obtain the symptoms’ significance based on BN’s determined EBP relationships. The FCM is a modeling approach with two main advantages: it is easily understandable by experts of a particular domain and gives values to causal maps based on qualitative opinions [22]. The uncertainty and vagueness commonly associated with opinions are medical data characteristics due to enormous individual differences and measurement errors. In general, the effectiveness of FCMs to deal with this variability has convinced researchers to implement this technique broadly. FCMs have been applied to make DSSs in settings where errors would have hazardous consequences [23] like thyroid diagnosis management [24], pulmonary differential diagnosis [25], estimate hospitals’ outputs level [26], and Acute Leukemia self-assessment DSS [27]. After determining the significance of symptoms in every severity level, the key symptoms for distinguishing the severity levels of confirmed cases of COVID-19 are used to implement in clinical trial measures on patients. For this study, a database from https://www.kaggle.com/ website has been collected, including COVID-19 symptoms for confirmed cases.

The rest of the paper is organized as follows: the implemented methods in this research are reviewed in "Methodology". The proposed approach is covered in "Proposed approach". In "Analysis of results", the results of the research are provided and analyzed. Finally, the conclusion of the study is discussed in "Conclusion".

## Methodology

This section is covered with the following materials: In "Introduction", the BN and its learning algorithm are introduced. In "Methodology", the mechanism of the FCM and its learning algorithm are provided.

### BNs and Bayesian search algorithm

Bayes theorem defines conditional or marginal probabilities for two variables $\alpha$ and $\beta$ as follows [21]:

1 $$ P(\alpha /\beta ) = \frac{P(\beta /\alpha )P(\alpha )}{{P(\beta )}}. $$

Human knowledge for multivariable problems can be considered a joint probability distribution (JPD) of these variables. Learning knowledge from data means to learn this joint probability. The joint probability has $2^{n}$ parameters for a binary variable problem with *n* variables. Obtaining the joint probability is unpractical, where the complexity of the problem increases exponentially with the number of variables. A BN decomposing the joint probability into the product of some simple conditional probabilities can decrease the complexity [28]. BNs are a member of the probabilistic graphical model family, which consists of nodes and directional arrows. Usually, nodes in a BN are depicted as circles or ovals and indicate variables, and directed edges (arrows) between pairs of nodes demonstrate relationships between variables. In the BNs, those nodes that contribute to higher nodes align themselves in “child”-to-“parent” relationships, where parent nodes are superior to the child nodes [29]. A BN has two components, a graphical model (*G*) and a set of parameters ($\Theta$) ($\beta = (G,\Theta \}$). *G* can be made from random variables $X_{1} ,X_{2} ,X_{3} ,...,X_{n}$ and $\Theta$ contains the states of each random variable given the parents set $\pi_{i}$ in *G*. In *G*, let random variables $V = \{ X_{1} ,X_{2} ,X_{3} ,...,X_{n} \}$ with JPD with their values or states $x_{1} ,x_{2} ,x_{3} ,...,x_{n}$ of *V*. The probabilities of these variables can be represented as $P(X_{1} = x_{1} ,\,\,X_{2} = x_{2} ,\,\,X_{3} = x_{3} ,\,\,...\,\,,\,\,X_{n} = x_{n} ) = P(x_{1} ,x_{2} ,x_{3} ,...x_{n} )$. A BN corresponds to graphical model *G*, which is a direct acyclic graph (DAG). The structure of DAG is described as vertices and directed edges. The vertices v is demonstrated as the set of nodes in *V*, and the edges denote the relationship between the vertices. Finally, each vertex in the graphical structure against *V* has its specific conditional probability distribution (CPD), which can be defined as $P(x_{i} |\pi_{i} )$. Therefore, the JPD of BN is the product of CPDs [30]. For more simplification, suppose that a BN consists of *n* random variables as $x_{1} ,x_{2} ,x_{3} ,...,x_{n}$. The full JPD can be written as follows [27, 31]:

2 $$ P(x_{1} ,x_{2} ,x_{3} ,...,x_{n} ) = P(x_{1} |x_{2} ,x_{3} ,...,x_{n} )P(x_{2} |x_{3} ,x_{4} ,...,x_{n} )...\,P(x_{n - 1} |x_{n} )P(x_{n} ). $$

Then, it can be simplified as:

3 $$ P(x_{1} ,x_{2} ,x_{3} ,...,x_{n} ) = \prod\limits_{i = 1}^{n} {P(x_{i} |x_{i + 1} ,...,x_{n} ).} $$

Afterward, suppose that $\pi_{i}$ indicates the set of parent nodes of the node $x_{i}$. Now, using the existing knowledge of what the parents of each node are, Eq. 3 can be reformulated as:

4 $$ P(x_{1} ,x_{2} ,x_{3} ,...,x_{n} ) = \prod\limits_{i = 1}^{n} {P(x_{i} |\pi_{i} } ). $$

Furthermore, BN assumes the independence assumption of nodes from its predecessors’ whole set apart from the direct parental [30].

The way of building an effective BN has been a long-term research issue. Building a Bayesian network for a given dataset *D* is finding the most appropriate network and generally is divided into two learning subtasks: structure learning and parameter learning. The structure learning seeks to determine the topology of the network. On the contrary, parameter learning concentrates on determining each conditional distribution $P(x_{i} |\pi_{i} )$ for a given network structure. Learning the BN structure requires higher accuracy rather than parameter learning [32]. The Bayesian search structure learning algorithm is one of the earliest and the most popular algorithms for BN. It was introduced by Cooper and Herskovits [33] and then was developed slightly by Heckerman et al. [34]. It follows essentially a hill-climbing procedure (guided by a scoring heuristic) with random restarts. The algorithm produces an acyclic directed graph that gives the maximum score. The score is proportional to the probability of the data given the structure, which assumes that the same prior probability has been assigned to any structure, which is proportional to the probability of the structure given the data. The algorithm generates an on-screen text box that consists of all parameters’ settings of the BS algorithms. The Bayesian search theory’s foundation is updating the probability of the located target in the $i_{th}$ box based on previous failures to detect. After searching the $i_{th}$ box and failing to find it, the likelihood of being located there will reduce, and the probability of being located in another box will augment. In this model, only the conditional probabilities of detection failure have a role in updating the new location probabilities. Consequently, only the box with the highest likelihood of containing the target and searches is being explored using the search algorithm. Upon failure to find, it updates all location probabilities and repeats until the target is detected or the number of glimpses has been reached [35]. The algorithm includes the following parameters: maximum parent count, algorithm iterations, the sample size for representing the inertia of the current parameters when introducing new data, a seed that is the initial random number, link probability that is a parameter used when generating a random starting network at the outset of each of the iterations, prior link probability, max time, and scoring function [36].

### FCM method

For the first time, Kosko [37] introduced the concept of FCM by utilizing fuzzy logic and artificial neural networks (ANN) tools to draw a cognitive map or the cause-and-effect graphical models that those cause-and-effect relations can acquire numbers in the range [0, 1] or [−1, 1] [38]. FCM can be created by time-series and experience and knowledge of experts in the subject [39]. In the FCM, $C_{i}$ demonstrates nodes or concepts which are connected via weighted arcs. Each connection between the two concepts $C_{i}$ and $C_{j}$ has a weight equal to $W_{ij}$, which indicates the degree of causality and the type of relationship between concepts [40]. So that $W_{ij} > 0$ represents a positive causal relationship, $W_{ij} < 0$ represents a negative causal relationship, and $W_{ij} = 0$ represents the absence of any relationship between the two concepts. After depicting the map, it must be modeled by mathematical formulas at the first step for analyzing the model. By achieving values of a node, the values of other nodes connected with this node can be obtained using Eq. 5:

5 $$ A_{i}^{(k + 1)} = f\left( {A_{i}^{(k)} + \sum\limits_{\begin{subarray}{l} i = 1 \\ j \ne i \end{subarray} }^{n} {W_{ij}^{(k)} A_{j}^{(k)} } } \right). $$

In Eq. 5, $A_{i}^{(k + 1)}$ indicates the value of $C_{i}$ in (*k* + *1*) repetitions, $A_{i}^{(k)}$ indicates the value of $C_{i}$ in *k* repetitions, and $f(x)$ represents the normalization function.

In the FCM, for increasing their accuracy, improving the map’s structure, and reducing reliance on experts’ opinions, precise estimation of the map weights by learning algorithms is indispensable [41]. FCMs’ learning algorithms are classified into four categories: Hebbian-based, population-based, hybrid algorithms, and other algorithms. Each category has corresponding characteristics and consists of several algorithms [42]. In the first category, Hebbian rule-based learning algorithms whose logic is derived from ANN, such as differential Hebbian [43], nonlinear Hebbian [44], and active Hebbian [45], have been developed. In the population-based learning algorithms, such as multi-local and balanced memetic algorithms [46], asexual reproduction optimization, and its modified version [47], etc., have been proposed. The third category of algorithms is based on both Hebbian-based and population-based algorithms, which can utilize human knowledge with historical data to adjust the weighting matrix. In the last category, developed algorithms are not in the main three groups and have been introduced to solve some of the previous algorithms’ problems, such as the Delta-rule algorithm [38, 48].

## Proposed approach

In this study, a self-assessment DSS is proposed to utilize the key symptoms of COVID-19 for distinguishing the severity of the epidemic based on the “Diagnosis and Treatment Protocol for Novel Coronavirus Pneumonia released by the National Health Commission (NHC) & State Administration of Traditional Chinese Medicine (NATCM) on 3 Mar. 2020” [49]. This method is a hybrid approach based on BNs and FCM. Initially, a set of data for confirmed cases of COVID-19 are collected, and after clustering data, they are categorized into three levels that exhibit the severity of the disease. In the first stage of this approach, the BN is implemented for two main purposes: (1) extracting the EBP relationships between attributes and their impact probability in the independent and combined modes; (2) extracting the EBP relationships between attributes inside the severity levels for developing FCM. First, the Bayesian search algorithm has been utilized to learn BN. This algorithm exploits background knowledge that can apply the experts’ opinion in the network, and this characteristic has been used in this study. In BN’s learning phase, relations between attributes are defined based on the conditional probability and the algorithm. The Bayesian search algorithm generates an acyclic directed graph that designates the maximum score. The score is proportional to the probability of the data given the structure. It considers that the same prior probability has been specified to any structure, proportional to the structure’s probability given the data. It should be mentioned that illogical relations between attributes are removed. For instance, fever cannot affect a patient’s age, and consequently, this relation should be eliminated.

**Fig. 3:**
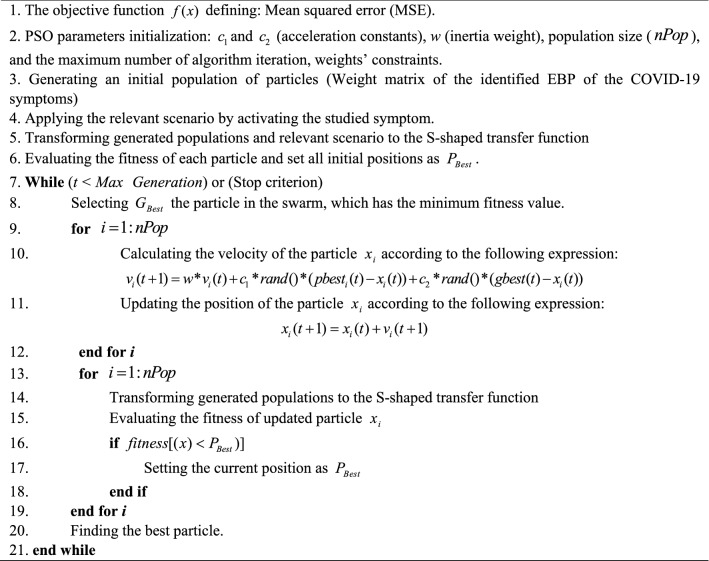
The pseudo-code of PSO-STF

**Fig. 4:**
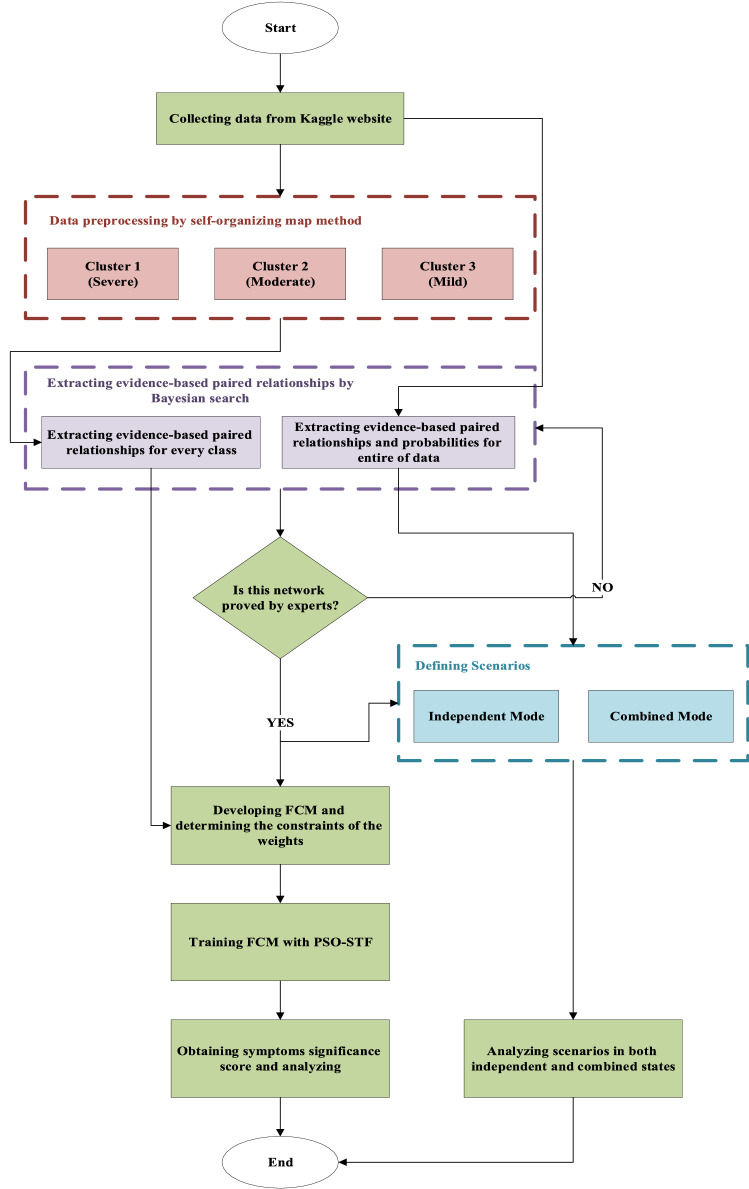
The flowchart of the proposed approach

In the second stage, the FCM is constructed based on the extracted EBP relationships by BN. The disease’s symptoms have been considered the main concepts of the FCM, and the severity of every level is the goal node of the FCMs. Developing FCM is based on the defining scenario for every symptom and achieving each symptom’s impact on the goal node. For this purpose, every symptom is activated, and the rest of the symptoms are deactivated, and the FCM is trained. After training FCM for every symptom and severity level, the goal node’s amount is picked out. In this study, due to the high importance of extracting the weights of EBP relationships between symptoms, a population-based learning algorithm has been utilized for training FCM. The used algorithm is based on the combination of the particle swarm optimization (PSO) and the S-shaped Transfer Function (PSO-STF). For the first time, this algorithm was proposed by Abbaspour Onari et al. [50] as an extension of the PSO algorithm due to its shortcoming in distinguishing between various concepts. The PSO-STF, implementing the S-shaped transfer function, can enhance the algorithm’s separability and give an accurate and vivid insight into the problem for decision-makers. It is practical in realms that concepts’ priority is crucial for decision-makers, and they need a precise and valid prioritization approach. Initially, the PSO generates various solutions for FCM, and due to generating solutions with high accuracy and separability, the S-shaped transfer function is applied to the algorithm. The corresponding weights between relations were allowed to assume values in the range [0, 1] to avoid generating physically meaningless weight matrices and lack of information about the impact of symptoms on each other. The pseudo-code for the mentioned algorithm is presented in Fig. 3. Finally, all of the achieved amounts of goal nodes in triple levels are picked out and are considered as the significance of symptoms in distinguishing the COVID-19 severity levels. It should be mention that symptoms with a higher score will have higher rankings. This approach has been elaborated in Fig. 4.

## Analysis of results

In this section, the results of the proposed approach are analyzed. This section is organized as follows: In the first section, the research’s preprocessing phase is presented for data clustering. In the second section, the proposed approach’s implementation is provided, and the results are analyzed.

### Data preprocessing for clustering

This study uses a dataset from https://www.kaggle.com/ website to be used as the COVID-19 dataset. In this dataset, the identified symptoms of COVID-19 have been considered as attributes. In this study, owing to the lack of genuine labels for samples, the clustering of these samples has been carried out using the self-organizing map. A self-organizing map, also known as the Kohonen map, belongs to instance-based ML algorithms. It can be visualized simply as a grid of neurons (nodes) of competitive nature where the outputs of these types of ANNs represent the network’s actual state [51]. It is one type of unsupervised learning ANN techniques. It is a simple and practical tool for clustering in data mining applications. In principle, data with similar features are divided into similar groups [52]. Overall, the self-organizing map consists of four main phases: initialization phase, competitive phase, cooperation phase, and adjustment phase [53]. To start clustering, each node is initialized with a random weight. According to a uniform distribution, this can be an arbitrary probability distribution or even randomly sampled from the input training set. The learning process is a set of iterations and is based on a simple rule: each node “competes” to be the best match to a randomly selected vector from the training set. The best matching unit (BMU) is rewarded by becoming more like the input vector. Moreover, nodes in the proximity of the BMU are also allowed to be altered in the same direction; however, to a lesser extent than the BMU. After a multitude of samplings, the nodes can learn to become more like the training set [54]. In this regard, all samples have been categorized into three clusters based on the self-organizing map with lattice size = [1, 3]. To put it differently, the distance between the center point of each cluster and the coordinate system’s origin has been considered a criterion to assign a real label to each cluster. Those labels are clinical severities defined by the NHC and NATCM protocols: (1) severe severity; (2) moderate severity; and (3) mild severity. So that 37.50% of studied samples have been placed in Cluster 1 entitled “severe severity” because of their greatest Euclidean distance from the origin of the coordinate system. In this cluster, an adult case has to meet one or more of the identified criteria, including “Respiratory distress”, “Oxygen saturation ≤ 93% at rest”, “Arterial partial pressure of oxygen”, and “having chest imaging that showed obvious lesion progression within 24–48 h > 50%”. Also, a child case of Cluster 1 can experience specific conditions, including “Tachypnea independent of fever and crying”, “Oxygen saturation ≤ 92% on finger pulse oximeter taken at rest”, “Labored breathing, cyanosis, and intermittent apnea,” “Lethargy and convulsion”, and “Difficulty feeding and signs of dehydration” [49]. Furthermore, the self-organizing map has categorized 18.75% and 43.75% of available samples in Cluster 2 entitled “moderate severity” and Cluster 3 entitled “mild severity”, respectively [49]. That is to say, if someone has fever and respiratory symptoms with radiological findings of pneumonia, they experience a moderate level of severity. The mild severity label indicates the cases experiencing a mild clinical symptoms level and no sign of pneumonia on imaging. After this stage, these clusters are considered as severity levels and are used as the input of BN and FCM.

### Implementing the proposed approach

This section is categorized into two sections. In "Introduction", the EBP relationships and impact probability of the symptoms are obtained in the independent and combined modes. In "Methodology", the EBP relationships between the symptoms into the triple severity level are extracted, and the FCM is constructed.

#### Assessment of scenarios

The collected data set consists of confirmed cases of COVID-19 patients. Data consists of nine symptoms, which are categorized into two sub-levels: (1) symptoms include fever, fatigue, dry cough, dyspnoea, and sore throat; (2) experiencing symptoms includes pains, nasal congestion, rhinorrhoea, and diarrhea. Both levels have a state that indicates none of the mentioned symptoms has been observed in the cases: none symptoms and none experiencing symptoms. Patients are persons between 5 age intervals: [0, 9], [10, 19], [20, 24], [25, 59], and upper 60. However, because age does not categorize as the symptoms of the epidemic and does not affect the decision-making process, it has not been studied in the scenarios.

**Fig. 5:**
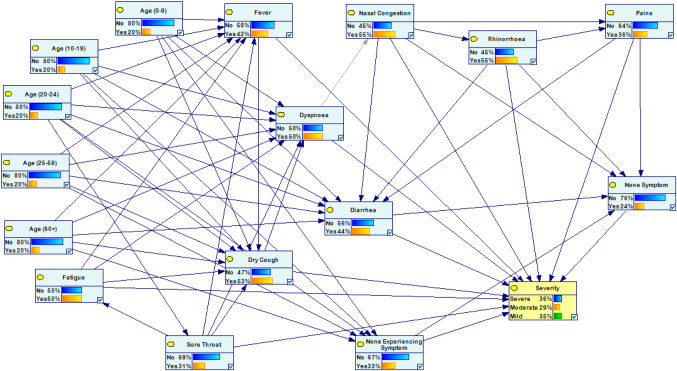
A general overview of COVID-19 symptoms’ evidence-based paired relationships

For executing BN, the Bayesian research and QGeNIe Modeler [55] method have been used. Figure 5 demonstrates the EBP relationships of the COVID-19 symptoms generated by BN from data.

In this phase, symptoms have been analyzed in two modes: (1) independent and (2) combined with different scenarios. Table 1 demonstrates the independent assessment mode based on BN, and probabilities have been extracted for triple severity levels. For illustration, in the symptoms’ category, a patient who has experienced fever, with a probability of 0.4243 could be categorized in severe severity. Because fever has a meaningful difference in the probability with the others, it has a key role in distinguishing the severity. On one side, a patient without any symptoms cannot easily classify in the severity levels, because all of the probabilities have a close range (0.33, 0.33, 0.34), and supplementary clinical trials should be considered for the patient to demonstrate the epidemic’s severity. This symptom cannot be effective in distinguishing the severity of the epidemic. In other words, although according to the official protocols, it is one of the main symptoms of the disease, it is not appropriate to be used to differentiate the levels of disease severity. This argument is true in the experiencing symptoms’ category when the role of rhinorrhoea is analyzed. For this symptom, the probabilities are (0.3625, 0.2799, and 0.3576) which role of this symptom in separating between severe severity and mild severity is controversial. The role of other symptoms can be analyzed in the same way.

**Table 1:** Evaluating the impact of disease symptoms on its severity in the independent mode

Row	Category	Studied symptom	Severity of illness		
			Severe (probability)	Moderate (probability)	Mild (probability)
1	Symptoms	Fever	0.4243	0.2257	0.3500
2		Fatigue	0.4558	0.2416	0.3026
3		Dry cough	0.4839	0.3416	0.1745
4		Dyspnoea	0.4589	0.2521	0.2890
5		Sore throat	0.4316	0.2043	0.3641
6		None symptom	0.3300	0.3300	0.3400
7	Experiencing symptoms	Pains	0.3615	0.2838	0.3547
8		Nasal Congestion	0.3618	0.2825	0.3557
9		Rhinorrhoea	0.3625	0.2799	0.3576
10		Diarrhea	0.3572	0.2897	0.3531
11		None Experiencing symptom	0.3351	0.3282	0.3367

Table 2 presents different scenarios for evaluating the symptoms based on BN. 18 scenarios have been designed to analyze confirmed cases’ potential severities. For instance, for a patient, if diarrhea symptom appears without any previous symptoms, determining the exact severity of his/her illness is very difficult, because all of the probabilities are very close to each other: (0.3306, 0.3306, and 0.3388). However, the probability of considering it as mild severity is more than others. Another scenario with the sore throat and not experiencing symptoms declares that distinguishing the disease’s severity again is difficult due to its range: (0.3407, 0.322, and 0.3371). Again, the probability of classifying this patient on the severe level is more than others. Diagnosing the disease’s severity without any previous disease symptoms is very difficult, because COVID-19 has very close mutual symptoms with other infectious diseases. As mentioned before, COVID-19 has some common symptoms (illustrated by CS in Table 2), which demonstrate in the first stages of the disease, and some later symptoms (showed by LS in Table 2) that exhibit themselves in the next stages of the disease. The combination of these symptoms may have a key role in distinguishing the disease. If a patient experiences fever, fatigue, and dry cough, he/she might suffer from severe severity of the disease with a probability of 0.7058. On one side, if an additional symptom observes in the case, like diarrhea, with the probability of 0.6824, he/she could experience the severe severity of the epidemic. An important point that should be taken into account is that, according to the independent mode, distinguishing the disease’s severity with diarrhea is very difficult due to its close probabilities (0.3572 for severe and 0.3531 for mild). Hence, it cannot have an important effect on distinguishing the severity of the disease. The rest of the scenarios in the same way according to the independent scenarios are analyzable.

**Table 2:** Evaluating the impact of disease symptoms on its severity in the combined mode

Row	Studied symptoms	Severity of illness		
		Severe (probability)	Moderate (probability)	Mild (probability)
1	None symptom + pains	0.3300	0.3300	0.3400
2	None symptom + nasal congestion	0.3297	0.3297	0.3406
3	None symptom + rhinorrhoea	0.3291	0.3291	0.3418
4	None symptom + diarrhea	0.3306	0.3306	0.3388
5	None symptom + all experiencing symptoms	0.3291	0.3291	0.3418
6	None Experiencing Symptom + Fever	0.3419	0.3214	0.3367
7	None experiencing symptom + fatigue	0.3461	0.3229	0.3310
8	None experiencing symptom + dry cough	0.3497	0.3360	0.3143
9	None experiencing symptom + dyspnoea	0.3458	0.3250	0.3292
10	None experiencing symptom + sore throat	0.3407	0.3222	0.3371
11	None experiencing symptom + all symptoms	0.3888	0.3056	0.3056
12	Fever + fatigue (CS)	0.5168	0.1806	0.3026
13	Fever + dry cough (CS)	0.5830	0.2085	0.2085
14	Fatigue + dry cough (CS)	0.6045	0.2484	0.1471
15	Fever + fatigue + dry cough (CS)	0.7058	0.1471	0.1471
16	Fever + fatigue + dry cough + diarrhea (CS&LS)	0.6824	0.1588	0.1588
17	Fever + fatigue + dry cough + sore throat (CS&LS)	0.6834	0.1583	0.1583
18	Fever + fatigue + dry cough + diarrhea + sore throat (CS&LS)	0.6658	0.1671	0.1671

#### Developing BN-based FCM

In this section, according to the clustered data in “Data preprocessing for clustering” section, the EBP relationships between symptoms of triple severities are obtained by BN and Bayesian search algorithm. In conventional FCMs, causal relationships between concepts are defined by human knowledge. However, in this study, EBPs for training FCM are determined automatically by BN. The PSO-STF algorithm has been implemented to train FCM based on the PSO algorithm and S-shaped transfer function. The algorithm achieves the weight of the relations between symptoms by optimizing them. The corresponding weights between algorithms are allowed to assume between [0, 1]. The maximum number of iterations and population size is set to 400 and 50, respectively, and the solution with the lowest fitness function has been collected for analysis. Clerc and Kennedy [56] generalized the PSO algorithm model, containing a set of coefficients to control the system’s convergence tendencies. Their approach is implemented in this study, and the rest of the PSO parameters are set based on Eq. 6. The constriction coefficients are $\phi_{1} = \phi_{2} = 2.05$ and $\Phi = \phi_{1} + \phi_{2}$. The value of $\chi$ is attained based on Eq. 6 and $\Phi$. The inertia weight $\omega$ is set to $\chi$. The acceleration coefficients, $c_{1}$ and $c_{2}$, are obtained as $c_{1} = \phi_{1} \times \chi$ and $c_{2} = \phi_{2} \times \chi$:

6 $$ \chi = \frac{2}{{\Phi - 2 + \sqrt {\Phi^{2} - 4\Phi } }}. $$

**Fig. 6:**
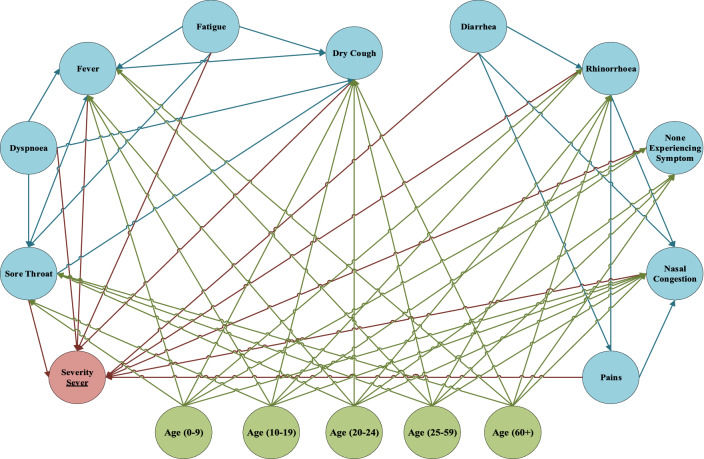
An overview of FCM and weights of EBP relationships inferred between symptoms for severe level

**Fig. 7:**
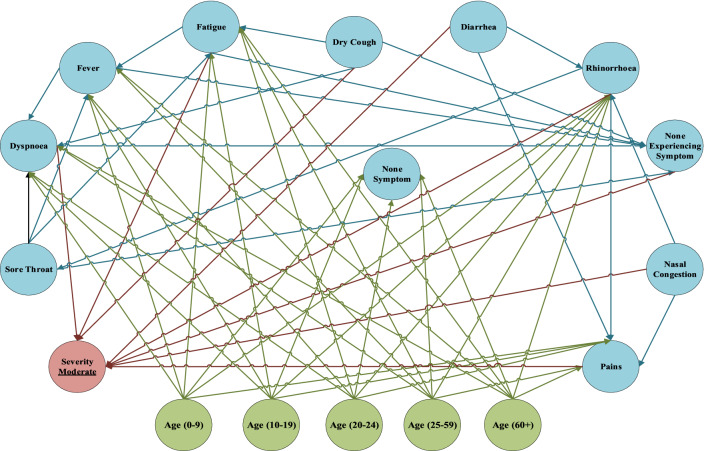
An overview of FCM and weights of EBP relationships inferred between symptoms for the moderate level

**Fig. 8:**
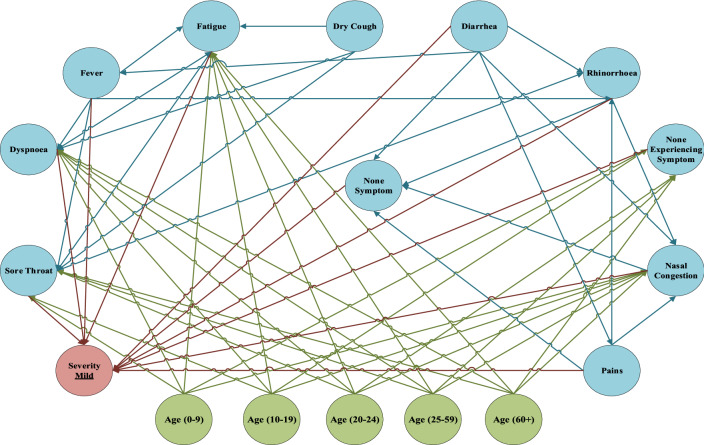
An overview of FCM and weights of EBP relationships inferred between symptoms for the mild level

Figures 6, 7, and 8 illustrate the structure of the FCM in triple severities based on the BN and automatic weighting of FCM.

After training FCM and reaching the steady state, the significance of symptoms in the triple severities is obtained. These significant scores are the main factors for separating new cases into the severity levels, and they are discriminators of diagnosing the severity of the disease. In Table 3, the significance of symptoms in the triple severity levels is illustrated and ranked based on their score. Dyspnoea, pains, and rhinorrhoea are the key factors for classifying new cases in severe severity. If a new confirmed case is experiencing dyspnoea, pains, and rhinorrhoea could categorize in severe severity. Sore throat, fever, and none experiencing symptoms cannot classify the patient effectively in severe severity. On one side, pains, fatigue, and dry cough can have a separable role in moderate severity. Those are the key element of classifying the patients in moderate severity. However, sore throat, fever, and none experiencing symptoms cannot discriminate symptoms in moderate severity. By analyzing none experiencing symptoms, sore throat, and diarrhea, which have the highest score in mild severity can classify new confirmed cases into mild severity. These symptoms have the highest priority in categorizing confirmed cases in mild severity. On the other hand, fever, non-symptoms, and nasal congestion cannot be counted as discriminator factors to classify mild severity cases.

**Table 3:** The significance of symptoms for classifying confirmed cases into triple severities

Row	Category	Studied symptom/active node	Level 1 (severe)		Level 2 (moderate)		Level 3 (mild)	
			Severity	Rank	Severity	Rank	Severity	Rank
1	Symptoms	Fever	0.5562	9	0.4865	10	0.5016	11
2		Fatigue	0.5292	10	0.5814	2	0.5662	7
3		Dry cough	0.5920	7	0.5579	3	0.5717	6
4		Dyspnoea	0.6350	1	0.5353	5	0.5383	8
5		Sore throat	0.5712	8	0.4806	11	0.6426	2
6		None symptom	0.6068	6	0.5321	6	0.5109	10
7	Experiencing Symptoms	Pains	0.6330	2	0.6097	1	0.5902	4
8		Nasal congestion	0.6214	4	0.5106	7	0.5350	9
9		Rhinorrhoea	0.6260	3	0.4994	8	0.5738	5
10		Diarrhea	0.6192	5	0.5368	4	0.5958	3
11		None experiencing symptom	0.5280	11	0.4978	9	0.6667	1

This study seeks the key symptoms of the disease, which can distinguish the severity of the COVID-19. However, based on the results, it does not necessarily mean that the epidemic’s most common symptoms have this characteristic. This self-assessment DSS can consider the EBP relationships that human and normal data cannot infer. In other words, without considering the relations between attributes, the key symptoms cannot be inferrable.

## Conclusion

Based on the WHO report, COVID-19 is a global outbreak that threatens all human beings’ lives on Earth. In the meantime, diagnosing the COVID-19 based on considerable errors of the main available methods, such as RT-PCR, has remained challenging to scientists. Due to the rapid spread of disease throughout the world, using a framework to categorize newly confirmed patients can help organize them effectively. This research aims to propose a self-assessment DSS to help classify new confirmed cases into triple severe severity. For this purpose, a dataset has been collected from confirmed cases, and they are analyzed as the whole and in the triple severity levels. For clustering data into severity levels, the self-organizing map method has been implemented. Then, BN is utilized for extracting EBP relationships between symptoms and their impact probability for analyzing scenarios and extracting EBP relationships inside the levels to develop FCM. Based on the defined scenarios, in the first phase of the research, the probability of the triple levels’ symptoms should have a reasonable interval for distinguishing the epidemic level. Without that, classifying the severities cannot reliably possible. Moreover, defining scenarios based on the combination of the symptoms can effectively impact the distinguishing levels. After developing FCM and reaching the steady state, the significance of symptoms is ranked based on their scores to study their potential in distinguishing the severity of the disease. Symptoms with higher scores have a key role in classifying the patients in the triple severity levels. Based on these scores, dyspnoea, pains, and rhinorrhoea have the main role in distinguishing severe severity. In the moderate severity, pains, fatigue, and dry cough can make the distinction between severities. Finally, for mild severity, none experiencing symptoms, sore throat, and diarrhea are considered as the key symptoms for distinguishing between severities of the COVID-19. The proposed self-assessment DSS can consider the EBP relationships between symptoms that cannot be inferable for humans. Results show that the common symptoms are not necessarily the key factors for distinguishing between severities, and relations between symptoms should be taken into account for analyzing them.

For future studies, it is suggested that to consider some of the disease’s new symptoms for analyzing their severity. Also, it is possible to utilize linguistic terms for reporting the severity of the symptoms and converting them to fuzzy numbers for conserving the accuracy of the information. It is worth suggesting that the proposed approach can be developed as an online application to enrich continuously with new and comprehensive data.

## References

[1] Ai T, Yang Z, Hou H, Zhan C, Chen C, Lv W, Tao Q, Sun Z, Xia L (2020). Correlation of chest CT and RT-PCR testing in Coronavirus Disease 2019 (COVID-19) in China: a report of 1014 cases. Radiology.

[2] Wang CJ, Ng CY, Brook RH (2020). Response to COVID-19 in Taiwan: big data analytics, new technology, and proactive testing. JAMA.

[3] Wang W, Tang J, Wei F (2020). Updated understanding of the outbreak of 2019 novel coronavirus (2019-nCoV) in Wuhan, China. J Med Virol.

[4] Hani C, Trieu NH, Saab I, Dangeard S, Bennani S, Chassagnon G, Revel MP (2020). COVID-19 pneumonia: a review of typical CT findings and differential diagnosis. Diagn Interv Imaging.

[5] Worldometer (2021) Daily reports of statistics about COVID-19. https://www.worldometers.info/coronavirus/

[6] Our World in Data (2021) Open source charts for COVID-19. https://ourworldindata.org/how-to-embed-charts

[7] Rothan HA, Byrareddy SN (2020). The epidemiology and pathogenesis of coronavirus disease (COVID-19) outbreak. J Autoimmun.

[8] Srinivasa Rao ASR, Vazquez JA (2020). Identification of COVID-19 can be quicker through artificial intelligence framework using a mobile phone-based survey when cities and towns are under quarantine. Infect Control Hosp Epidemiol.

[9] Li L, Qin L, Xu Z, Yin Y, Wang X, Kong B, Bai J, Lu Y, Fang Z, Song Q, Cao K, Liu D, Wang G, Xu Q, Fang X, Zhang S, Xia J, Xia J (2020). Using artificial intelligence to detect COVID-19 and community-acquired pneumonia based on pulmonary CT: evaluation of the diagnostic accuracy. Radiology.

[10] Huang Z, Zhao S, Li Z, Chen W, Zhao L, Deng L, Song B (2020). The Battle Against Coronavirus Disease 2019 (COVID-19): Emergency Management and Infection Control in a Radiology Department. J Am Coll Radiol.

[11] Xu X, Han M, Li T, Sun W, Wang D, Fu B, Zhou Y, Zheng X, Yang Y, Li X, Zhang X, Pan A, Wei H (2020). Efective treatment of severe COVID-19 patients with tocilizumab. Proc Natl Acad Sci USA.

[12] Tang Z, Zhao W, Xie X, Zhong Z, Shi F, Liu J, Shen D (2020) Severity Assessment of Coronavirus Disease 2019 (COVID-19) using quantitative features from chest CT images. http://arxiv.org/abs/2003.11988

[13] Farid AA, Selim GI, Awad H, Khater A (2020). A novel approach of CT images feature analysis and prediction to screen for corona virus disease (COVID-19). Int J Sci Eng Res.

[14] Karar ME, Hemdan EE-D, Shouman MA (2020). Cascaded deep learning classifiers for computer-aided diagnosis of COVID-19 and pneumonia diseases in X-ray scans. Complex Intell Syst.

[15] Shankar K, Perumal E (2020). A novel hand-crafted with deep learning features based fusion model for COVID-19 diagnosis and classification using chest X-ray images. Complex Intell Syst.

[16] Elaziz MA, Hosny KM, Salah A, Darwish MM, Lu S, Sahlol AT (2020). New machine learning method for imagebased diagnosis of COVID-19. PLoS ONE.

[17] Laguarta J, Hueto F, Subirana B (2020). COVID-19 artificial intelligence diagnosis using only cough recordings. IEEE Open J Eng Med Biol.

[18] Chockalingam S, Pieters W, Teixeira A, van Gelder P (2017) Bayesian network models in cyber security: a systematic review. In: Lecture notes in computer science (including subseries lecture notes in artificial intelligence and lecture notes in bioinformatics), vol 10674, pp 105–122. LNCS, Springer. 10.1007/978-3-319-70290-2_7

[19] Kahn CE, Roberts LM, Shaffer KA, Haddawy P (1997). Construction of a Bayesian network for mammographic diagnosis of breast cancer. Comput Biol Med.

[20] Pinheiro PR, De Castro AKA, Pinheiro MCD (2008) A multicriteria model applied in the diagnosis of Alzheimer’s disease: A Bayesian network. In: Proceedings—2008 IEEE 11th International Conference on Computational Science and Engineering, CSE 2008. IEEE, pp 15–22. 10.1109/CSE.2008.44

[21] Özçift A, Gülten A (2013). Genetic algorithm wrapped Bayesian network feature selection applied to differential diagnosis of erythemato-squamous diseases. Digit Signal Process Rev J.

[22] Bakhtavar E, Aghayarloo R, Yousefi S, Hewage K, Sadiq R (2019). Renewable energy based mine reclamation strategy: a hybrid fuzzy-based network analysis. J Clean Prod.

[23] Giabbanelli PJ, Torsney-Weir T, Mago VK (2012). A fuzzy cognitive map of the psychosocial determinants of obesity. Appl Soft Comput J.

[24] Papageorgiou EI, Papandrianos NI, Apostolopoulos DJ, Vassilakos PJ (2008) Fuzzy cognitive map based decision support system for thyroid diagnosis management. In IEEE International Conference on Fuzzy Systems. IEEE, pp1204–1211. 10.1109/FUZZY.2008.4630524

[25] Bourgani, E., Stylios, C. D., Manis, G., & Georgopoulos, V. C. (2014). Time dependent fuzzy cognitive maps for medical diagnosis. In: Lecture notes in computer science (including subseries lecture notes in artificial intelligence and lecture notes in bioinformatics), vol 8445, pp 544–554. LNCS, Springer. 10.1007/978-3-319-07064-3_47

[26] Rezaee MJ, Yousefi S, Hayati J (2018). A decision system using fuzzy cognitive map and multi-group data envelopment analysis to estimate hospitals’ outputs level. Neural Comput Appl.

[27] Rezaee MJ, Sadatpour M, Ghanbari-Ghoushchi N, Fathi E, Alizadeh A (2020). Analysis and decision based on specialist self-assessment for prognosis factors of acute leukemia integrating data-driven Bayesian network and fuzzy cognitive map. Med Biol Eng Comput.

[28] Zeng L, Ge Z (2020). Improved Population-Based Incremental Learning of Bayesian Networks with partly known structure and parallel computing. Eng Appl Artif Intell.

[29] Mittal A, Kassim A (eds) (2007) Bayesian network technologies: applications and graphical models: applications and graphical models. IGI Global

[30] Friedman N, Geiger D, Goldszmidt M (1997). Bayesian network classifiers. Mach Learn.

[31] Zhang X, Mahadevan S (2020). Bayesian network modeling of accident investigation reports for aviation safety assessment. Reliab Eng Syst Saf.

[32] Tan X, Gao X, Wang Z, He C (2020). Bidirectional heuristic search to find the optimal Bayesian network structure. Neurocomputing.

[33] Cooper GF, Herskovits E (1992). A Bayesian method for the induction of probabilistic networks from data. Mach Learn.

[34] Heckerman D, Geiger D, Chickering DM (1995). Learning Bayesian networks: the combination of knowledge and statistical data. Mach Learn.

[35] Kalkwarf B (2017) Search parameter optimization for discrete, Bayesian, and continuous search algorithms. Naval Postgraduate School Monterey United States

[36] GeNIe (2018) The Bayesian search algorithm description by GeNIe software. https://support.bayesfusion.com/docs/GeNIe

[37] Kosko B (1986). Fuzzy cognitive maps. Int J Man Mach Stud.

[38] Rezaee MJ, Yousefi S, Babaei M (2017). Multi-stage cognitive map for failures assessment of production processes: an extension in structure and algorithm. Neurocomputing.

[39] Rezaee MJ, Yousefi S, Valipour M, Dehdar MM (2018). Risk analysis of sequential processes in food industry integrating multi-stage fuzzy cognitive map and process failure mode and effects analysis. Comput Ind Eng.

[40] Alizadeh A, Yousefi S (2019). An integrated Taguchi loss function–fuzzy cognitive map–MCGP with utility function approach for supplier selection problem. Neural Comput Appl.

[41] Abbaspour Onari M, Jahangoshai Rezaee M (2020). A fuzzy cognitive map based on Nash bargaining game for supplier selection problem: a case study on auto parts industry. Oper Res Int J.

[42] Bakhtavar E, Valipour M, Yousefi S, Sadiq R, Hewage K (2020). Fuzzy cognitive maps in systems risk analysis: a comprehensive review. Complex Intell Syst.

[43] Dickerson JA, Kosko B (1994). Virtual worlds as fuzzy cognitive maps. Presence Teleoperators Virtual Environ.

[44] Papageorgiou E, Stylios C, Groumpos P (2003) Fuzzy cognitive map learning based on nonlinear hebbian rule. In: Lecture notes in computer science (including subseries lecture notes in artificial intelligence and lecture notes in bioinformatics), vol 2903. Springer, pp 256–268. 10.1007/978-3-540-24581-0_22

[45] Papageorgiou EI, Stylios CD, Groumpos PP (2004). Active Hebbian learning algorithm to train fuzzy cognitive maps. Int J Approx Reason.

[46] Salmeron JL, Ruiz-Celma A, Mena A (2017). Learning FCMs with multi-local and balanced memetic algorithms for forecasting industrial drying processes. Neurocomputing.

[47] Salmeron JL, Mansouri T, Moghadam MRS, Mardani A (2019). Learning fuzzy cognitive maps with modified asexual reproduction optimisation algorithm. Knowl Based Syst.

[48] Yousefi S, Jahangoshai Rezaee M, Moradi A (2020). Causal effect analysis of logistics processes risks in manufacturing industries using sequential multi-stage fuzzy cognitive map: a case study. Int J Comput Integr Manuf.

[49] National Health Commission (2020). Diagnosis and treatment protocol for novel coronavirus pneumonia (Trial Version 7). Chin Med J (Engl).

[50] Abbaspour Onari M, Yousefi S, Jahangoshai Rezaee M (2020). Risk assessment in discrete production processes considering uncertainty and reliability: Z-number multi-stage fuzzy cognitive map with fuzzy learning algorithm. Artif Intell Rev.

[51] Almi’Ani M, Ghazleh AA, Al-Rahayfeh A, Razaque A (2018) Intelligent intrusion detection system using clustered self organized map. In: 2018 5th International Conference on Software Defined Systems, SDS 2018. Institute of Electrical and Electronics Engineers Inc., pp 138–144. 10.1109/SDS.2018.8370435

[52] Rezaee MJ, Eshkevari M, Saberi M, Hussain O (2021). GBK-means clustering algorithm: An improvement to the K-means algorithm based on the bargaining game. Knowl Based Syst.

[53] Dadkhah M, Rezaee MJ, Chavoshi AZ (2018). Short-term power output forecasting of hourly operation in power plant based on climate factors and effects of wind direction and wind speed. Energy.

[54] Geach JE (2012). Unsupervised self-organized mapping: a versatile empirical tool for object selection, classification and redshift estimation in large surveys. Mon Not R Astron Soc.

[55] QGeNIe Modeler (2020) User Manual (n.d.)

[56] Clerc M, Kennedy J (2002). The particle swarm-explosion, stability, and convergence in a multidimensional complex space. IEEE Trans Evol Comput.

[57] de Moraes Batista AF, Miraglia JL, Donato THR, Chiavegatto Filho ADP (2020) COVID-19 diagnosis prediction in emergency care patients: a machine learning approach. medRxiv

[58] Wang C, Deng R, Gou L, Fu Z, Zhang X, Shao F, Wang G, Fu W, Xiao J, Ding X (2020). Preliminary study to identify severe from moderate cases of COVID-19 using combined hematology parameters. Ann Transl Med.

